# Genetic ancestry inferred from autosomal and Y chromosome markers and HLA genotypes in Type 1 Diabetes from an admixed Brazilian population

**DOI:** 10.1038/s41598-021-93691-x

**Published:** 2021-07-08

**Authors:** Rossana Santiago de Sousa Azulay, Luís Cristóvão Porto, Dayse Aparecida Silva, Maria da Glória Tavares, Roberta Maria Duailibe Ferreira Reis, Gilvan Cortês Nascimento, Sabrina da Silva Pereira Damianse, Viviane Chaves de Carvalho Rocha, Marcelo Magalhães, Vandilson Rodrigues, Paulo Ricardo Vilas Boas Carvalho, Manuel dos Santos Faria, Marília Brito Gomes

**Affiliations:** 1 Service of Endocrinology, University Hospital of the Federal University of Maranhão (HUUFMA/EBSERH), Rua Barão de Itapary, 227, Centro, São Luís, Maranhão 65020-070 Brazil; 2 Research Group in Clinical and Molecular Endocrinology and Metabology (ENDOCLIM), São Luís, Brazil; 3 Histocompatibility and Cryopreservation Laboratory (HLA), Rio de Janeiro State University (UERJ), Rio de Janeiro, Rio de Janeiro Brazil; 4 DNA Diagnostic Laboratory (LDD), Rio de Janeiro State University (UERJ), Rio de Janeiro, Rio de Janeiro Brazil; 5 Clinical Research Center of the University Hospital of the Federal University of Maranhão (CEPEC – HUUFMA), São Luís, Brazil; 6 Diabetes Unit, State University of Rio de Janeiro (UERJ), Rio de Janeiro, Brazil

**Keywords:** Genetics, Endocrinology

## Abstract

This study aimed to investigate the relationship between genetic ancestry inferred from autosomal and Y chromosome markers and HLA genotypes in patients with Type 1 Diabetes from an admixed Brazilian population. Inference of autosomal ancestry; *HLA-DRB1*, *-DQA1* and *-DQB1* typifications; and Y chromosome analysis were performed. European autosomal ancestry was about 50%, followed by approximately 25% of African and Native American. The European Y chromosome was predominant. The HLA-DRB1*03 and DRB1*04 alleles presented risk association with T1D. When the Y chromosome was European, DRB1*03 and DRB1*04 homozygote and DRB1*03/DRB1*04 heterozygote genotypes were the most frequent. The results suggest that individuals from Maranhão have a European origin as their major component; and are patrilineal with greater frequency from the R1b haplogroup. The predominance of the HLA-DRB1*03 and DRB1*04 alleles conferring greater risk in our population and being more frequently related to the ancestry of the European Y chromosome suggests that in our population, the risk of T1D can be transmitted by European ancestors of our process miscegenation. However, the Y sample sizes of Africans and Native Americans were small, and further research should be conducted with large mixed sample sizes to clarify this possible association.

## Introduction

Type 1 Diabetes (T1D) is a disorder of glucose homeostasis, which develops as a result of the synergistic effects of genetic, immunological, and environmental factors that lead to the loss of β cell secretory function^1,2^. The greatest genetic risk factor associated with T1D is in the histocompatibility leukocyte antigen system (HLA), especially in class II molecules, HLA-DR, and HLA-DQ^3,4^. Certain combinations of alleles are found in specific haplotypes of the *HLA-DRB1* ~ *DQA1* ~ *DQB1* genes, varying between some ethnic groups, conferring susceptibility or protection to the disease^3–5^. In Europeans, the greatest risk to the disease is associated with the haplotypes DRB1*04:01/02/04/05 ~ DQA1*03 ~ DQB1*03:02 and DRB1*03:01 ~ DQA1* 05:01 ~ DQB1*02:01^1,6,7^. In patients of African origin, additional haplotypes are associated with risk, such as DRB1*09:01 ~ DQA1*03:01 ~ DQB1*02:01 and DRB1*07:01 ~ DQA1*03:01 ~ DQB1*02:01^8^. It is also important to note that same haplotype, such as DR7 molecule, is often seen in Europeans with a protective effect and as a risk factor in African-American populations for T1D, being the most frequent protective haplotype in a study of Brazilian T1D patients, regardless of self-reported color-race (CRsr)^9^.

Advances in the study of genetic ancestry have brought an important contribution to the understanding of the processes of migration and colonization of peoples^10,11^. Usually, genomic markers are used to analyze individual ancestry, while single-parent markers of mitochondrial DNA (mtDNA) and Y chromosome are useful to understand human ancestral history^12,13^. The non-recombining portion of the Y chromosome (NRY) does not undergo recombination with the X chromosome during meiosis and is transmitted practically intact through the paternal strains as haplotypes^14^; the evaluations of their polymorphisms being useful for ancestral population analyzes^15^.

Brazil had its population formed through miscegenation between European, African, and Native American ethnicities. The mixture between these races was different in each Brazilian region, generating a highly heterogeneous population^10,16,17^. The state of Maranhão, located in the northeast region, historically suffered interference initially in the formation of its population of Europeans (Portuguese, French, and Dutch) Africans (Banto and Yoruba), and Native Americans^18–21^.

In almost all Brazilian populations, an asymmetric mating pattern usually occurs, mainly between European men and Native American or African women^6^. However, in Maranhão, it also occurred between African men and Native American women^22^, and Native American men with African or admixed women^23^. Thus, in Maranhão, three asymmetric mating models were identified.

Several studies have been conducted in Brazil in order to determine the population's autosomal ancestry and its regional differences^10–12,17,24,25^; and also the ancestry of the paternal lineage through Y-STR (Y- chromosomal Short Tandem Repeats) and Y-SNP (Y- chromosomal Single Nucleotide Polymorphism) markers; however, there is little information regarding the population of Maranhão^26–29^. Our study aimed to analyze the pattern of autosomal and Y chromosome ancestry in the general population and in patients with T1D in the state of Maranhão and to evaluate these findings with the genetic profile of HLA class II in these patients.

## Materials and methods

### Study design and samples

This is a cross-sectional study carried out at the Endocrinology Service of University Hospital of Federal University of Maranhão (HUUFMA), a tertiary service for the care of patients with T1D in São Luís, Maranhão State, Brazil. This study was approved by the ethics committee of the HUUFMA under opinion number 59795116.9.0000.5086. All participants or their legal representatives were informed about the objectives and procedures of the study and signed an informed consent form. We confirm that all experiments were performed in accordance with relevant guidelines and regulations.

The T1D patients above 10 years of age (n = 152) were recruited at the HUUFMA according to classic clinical criteria, such as polyuria, polydipsia, polyphagia, and weight loss associated with the need for insulin therapy since diagnosis. The control group (n = 286) was recruited from the blood bank of the Hematology Center of Maranhão (Hemomar). The controls were in good health, without diabetes or clinical or laboratory evidence of hepatitis B and C, AIDS (Acquired Immunodeficiency Syndrome), diseases associated with HTLV (Human T Lymphotropic Vírus) I and II viruses, Chagas disease, malaria, and Parkinson’s disease. Both Health Units are part of the National Brazilian Health System. The recruitment was performed at both Public Health Units from October 2016 to July 2018.

### Data collection

A semi-structured questionnaire was applied to collect demographic variables including sex, age (years), self-reported color/race and informed ancestry of family members (parents and grandparents) based on the IBGE ( Brazilian Institute of Geography and Statistics) classification: black = *preta,* white = *branca*, brown = *parda*, asian = *amarela* and indigenous = *indígena*^30^. Additionally, data about age at T1D diagnosis (years), duration of diabetes (years), body mass index (BMI) (kg/m^2^), and insulin daily dose (U/kg) were collected from T1D patients. The serum laboratory evaluation was determined by fasting blood glucose (enzymatic), and glycated hemoglobin A1c (HPLC—High Performance Liquid Chromatography) in T1D patients.

DNA extraction was performed on a peripheral blood sample using the SP QIA Symphony commercial kit according to the manufacturer's guidelines (Qiagen, USA).

### Genetic ancestry analysis

For inference of autosomal ancestry, a panel of 46 autosomal informational insertion/deletion ancestry markers (AIM–Indels) was used, amplified in a single multiplex PCR (Polimerase Chain Reaction) according to the protocol described by Pereira et al.^31^. The detection of polymorphisms in the generated fragments was performed by capillary electrophoresis in the ABI 3500 automatic sequencer (Life Technologies). Genotyping was performed by two independent analysts using GeneMapper Analysis Software v.4.1 (Life Technologies), and the results were compared for consistency. Structure v.2.3.3 software was used to estimate ancestry and the HGDP–CEPH (Human Genome Diversity Genotype Database- Centre d’Étude du Polymorphisme Humain) panel was used as a reference for ancestral populations^31^. The allele frequencies of 46 genotyped AIM–INDELs were compared with a database of a healthy Brazilian population for the same markers from all geographic regions of Brazil^10^.

We performed the analysis of the 26 STR markers of the Y chromosome belonging to the commercial kit Yfiler® Plus (Thermo Fisher Scientific Inc), amplified in a single multiplex PCR and followed by capillary electrophoresis, according to the manufacturer's protocol. The analysis of the amplification products and the naming of the alleles were performed using the GeneMapper Analysis Software v.4.1 (Life Technologies), when comparing the detected alleles with the alleles of the Yfiler® Plus Allelic Ladder. The determination of haplogroups was performed using two freely available software—Haplogroup Predictor (http://www.hprg.com/hapest5/)^32^ and NevGen Y–DNA Haplogroup Predictor (https://www.nevgen.org/)—based^33^ on the set of Y–STR haplotypes from the results obtained in Yfiler® Plus.

### HLA typings

The typification of the *HLA-DRB1*, *-DQA1*, and *-DQB1* genes was performed with the PCR–RSSO (high-resolution LabTypt (One Lambda Inc., West Hills, USA), combined with Luminex technology of T1D 152 patients and 75 from the 286 controls. The allelic definition was based on version 3.0 of the CWD (Common and Well Documented) list, and the ambiguities were resolved by sequencing methods^34^.

We also included information on *HLA-DRB1*, *-DQA1*, and -*DQB1* typings from 620 REDOME (National Registry of Bone Marrow Donors—Brazil) entries from the same Northeast Brazil region and matched for CRsr at a 4:1 ratio (Control/BMD—Bone Marrow Donors group). Inclusion criteria as a donor at REDOME are 18 to 55 years of age; good health status; and no infection, hematological, or immunological disease. Individuals who have had a diagnosis of cancer or diabetes with the use of insulin or other injectable medication are also excluded from REDOME^35^.

### Statistical analysis

The data analysis was performed using SPSS software version 26.0 (IBM, Chicago, IL, USA). Initially, descriptive statistics were performed by calculating measures of frequency, central tendency (mean and median), and dispersion (standard deviation, SD, and interquartile range; IQR). The normality of quantitative variables was assessed using the Shapiro-Wilk test. After this procedure, independent student’s t-test and one-way ANOVA post hoc Bonferroni were selected for the comparative analysis of continuous variables. Categorical variables were analyzed using the chi-square test, Fisher’s exact test, and chi-square with Bonferroni correction. Odds ratio (OR) and 95% confidence interval (95% CI) were used to determine the association. Also, Triplot software version 4.1.2 was used to build the diagrams of the genotypic ancestry profile. A network analysis between Y-chromosome haplogroups and HLA-DRB1* alleles was conducted using Gephi 0.9.2. The level of significance adopted for all analyses was 5%.

In addition, the software Minitab 19 (Minitab, LLC., State College, PA, USA) was used to perform ancestry-specific principal component analysis (ASPCA) on phased haploid genomes of 46 INDEL panel markers. ASPCA was performed in order to infer the sub-continental origin of the studied population on the basis of autosomal marker data. To run ASPCA on the geographical reference groups, we combined our Maranhão admixed individuals with the African 1 (Angola, Botswana, Namibia, South Africa, Lesotho, Central African Republic, Congo, Kenya) African 2 (Senegal and Nigeria), European (France, Italy, Russia, Orkney Islands), Mexico (Maya and Pima), Colombia and Brazilian Native American data sets (Karitiana, Surui, Santa Isabel and Terena). To run ASPCA on the Brazilian reference groups, we combined our Maranhão admixed individuals with the Brazilian Native American (Karitiana, Surui, Santa Isabel and Terena); and urban samples from Center-West (Mato Grosso do Sul), North (Amazonas), Northeast (Alagoas and Pernambuco), South (Paraná, Rio Grande do Sul, and Santa Catarina), and Southeast (Espírito Santo, Minas Gerais, Rio de Janeiro, and São Paulo).

## Results

### Overview of the study sample

Table 1 shows the distribution and the comparative analysis of demographic and clinical data. The control group was older and had a higher frequency of blacks than individuals with T1D.

**Table 1:** Demographic and clinical data of the studied population.

Variable	T1D (n = 152)	Control (n = 286)	*P* value
**Demographic data**			
Gender, male [n (%)]	79 (52.0)	146 (51.0)	0.853
Age in years [mean ± sd]	25.1 ± 10.6	32.9 ± 10.2	< 0.001
Self-reported color-race* [n (%)]			0.001
White	44 (29.0)	63 (22.0)	
Black	9 (5.9)	54 (18.9)	
Brown	99 (65.1)	167 (58.4)	
Indigenous	0 (0)	2 (0.7)	
Duration of diabetes (years)	11.3 ± 8.1		
Age at Diabetes Diagnosis (years)	13.7 ± 8.7		
Body mass index (kg/m^2^)	22.6 ± 7.5		
**Laboratorial data**			
HbA1c (%)	9.0 ± 2.3		
Insulin dose (UI\Kg)	0.8 ± 0.3		

Data are presented as number (percentage), mean ± standard deviation.

*T1D* type 1 diabetes, *HbA1c* hemoglobin A1c.

The comparative analysis of the color/race of family members reported by T1D and control groups showed a higher frequency of blacks in the control group for the mother's CRsr (15.7% versus 8.6% *P* = 0.02). These data are described in Supplementary Table 1S online.

### Analysis of autosomal and Y chromosome ancestry

**Figure 1:**
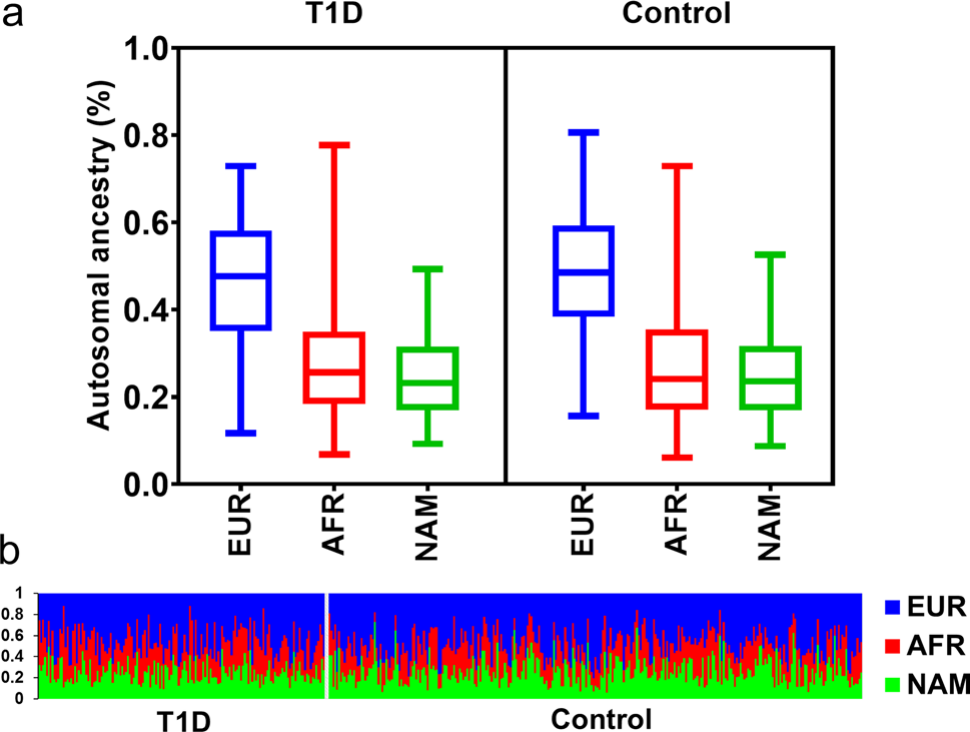
Box plot (**a**) and individual (**b**) proportions of the ancestry estimates for the patients with T1D and control group, using 46 AIM-Indel. *EUR* European. *AFR* African. *NAM* Native American. Ancestry estimates were obtained using STRUCTURE, for the following options: k = 3; 50,000 burning steps, followed by 50,000 Markov Chain Monte Carlo iterations; Admixture model (Use population Information to test for migrants); and allele frequencies were correlated and updated using only individuals with POPFLAG = 1.

No significant difference was detected in the proportion of autosomal ancestry between T1D and controls. European ancestry was the highest in both groups, followed by Africans and Native American in equal proportions. The distribution of autosomal ancestry is illustrated in Fig. 1. The European Y chromosome ancestry predominated in both groups. The proportions of autosomal and Y chromosome ancestry in T1D and controls are shown in Table 2.

**Table 2:** Distribution of ancestry markers of the Maranhão state T1D and control groups.

Ancestry markers	T1D (n = 152)	Control (n = 286)	*P* value
**Autosomal ancestry**			
European	47.3 [35.1–72.9]	48.5 [38.4–59.3]	0.147
African	25.6 [18.4–34.8]	24.1 [17.1–35.5]	0.125
Native American	23.1 [16.9–31.5]	23.5 [16.9–31.7]	0.891
**Y chromosome (n (%))**			0.443
European	67 (84.8)	124 (87.3)	
African	7 (8.9)	14 (9.9)	
Native American	5 (6.3)	4 (2.8)	

Data are presented as number (percentage), median [IQR].

*T1D* type 1 diabetes.

**Figure 2:**
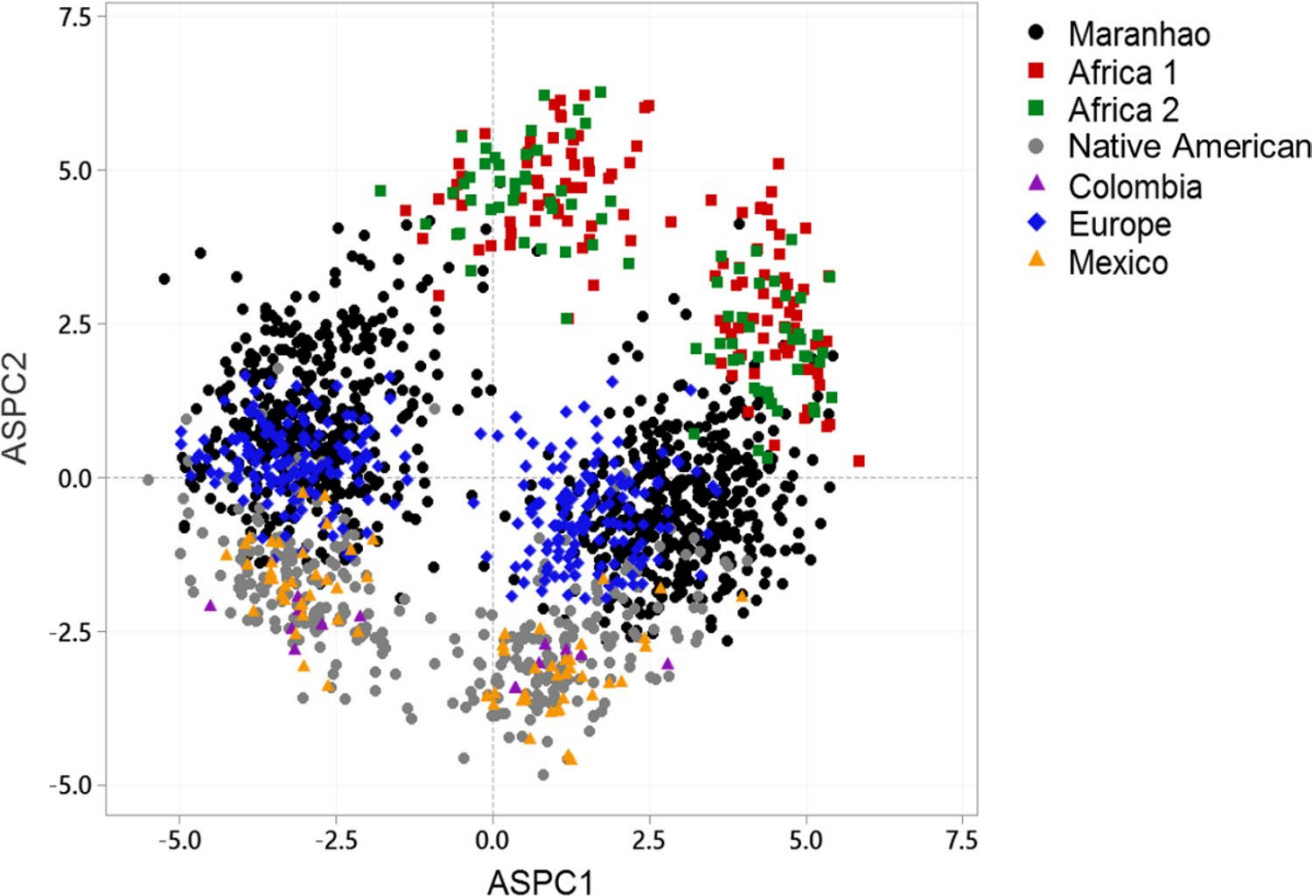
Ancestry specific by principal components analysis of admixed individuals and geographical reference groups. Africa 1 (Angola, Botswana, Namibia, South Africa, Lesotho, Central African Republic, Congo, Kenya), Africa 2 (Senegal Nigeria) Europe (France, Italy, Russia, Orkney Islands), Mexico (Maya and Pima), Brazilian Native American (Karitiana, Surui, Santa Isabel and Terena).

**Figure 3:**
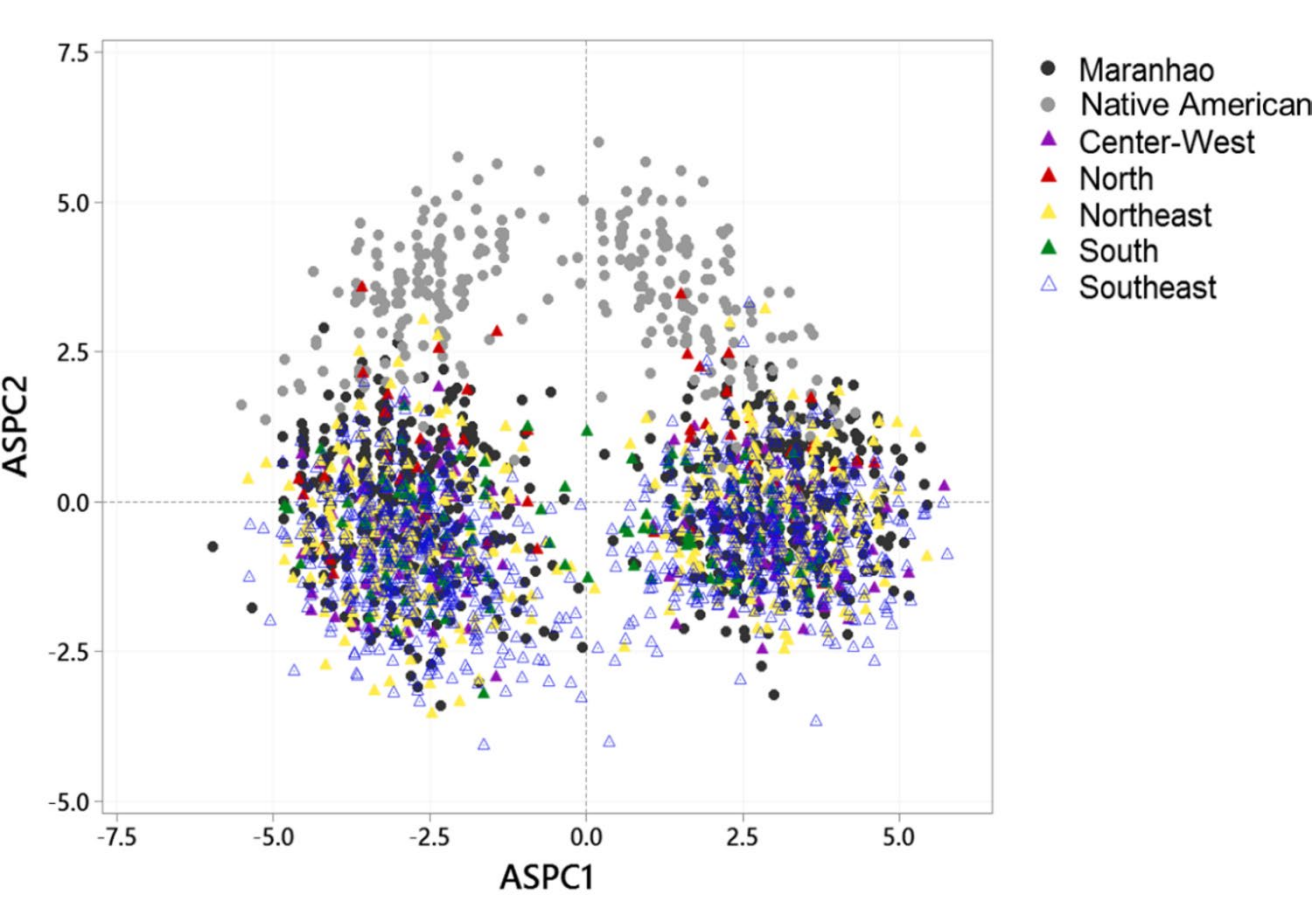
Ancestry specific by principal components analysis of admixed individuals and Brazilian reference groups. Brazilian Native American (Karitiana, Surui, Santa Isabel and Terena), and urban samples from Center-West (Mato Grosso do Sul), North (Amazonas), Northeast (Alagoas and Pernambuco), South (Paraná, Rio Grande do Sul, and Santa Catarina), and Southeast (Espírito Santo, Minas Gerais, Rio de Janeiro, and São Paulo).

Figure 2 shows the ancestry specific of the complete sample from Maranhão combined with European, Latin American, African, and Brazilian Native American data sets. The analysis revealed that the samples from Maranhão were heterogeneous. Most of the samples from Maranhão are grouped with Europeans, and Maranhão clustered closer to Native Americans than to Africans. Exploring the specific ancestry in Brazilian haploid samples, the ASPCA revealed that the Brazilian samples cluster into Native American and urban groups. The total sample from Maranhão was similar to other Brazilian urban agglomerations and showed haploid samples closer to Native American (Fig. 3).

Supplementary Fig. 1S online expresses the distribution of autosomal ancestry according to the self-reported color-race in the two study groups. In both groups (T1D and control) European ancestry was predominant among the self-reported whites and browns (*P* < 0.05). Also, a statistically higher proportion of African ancestry was observed in patients with T1D who self-reported as blacks (*P* < 0.05), as was a higher proportion of African and European ancestry (*P* < 0.05) among self-reported as black in the control group.

In the analysis of the Y chromosome haplogroups, we observed that R1b was the most frequent in both groups, followed by E1b1b in the TD1 group and by E1b1a in the control group, as shown in Table 3.

**Table 3:** Y-chromosome haplogroups of the Maranhão state T1D and control groups.

Haplogroup predictor	T1D (n = 79)		Control (n = 143*)	
	n	%	n	%
E total	21	26.58	24	16.78
E1a	1	1.27	0	0.00
E1b1a	6	7.59	14	**9.79**
E1b1b	14	**17.72**	10	6.99
G2a	5	6.33	4	2.80
I total	7	8.86	15	10.49
I1	2	2.53	4	2.80
I2a	5	6.33	9	6.29
I2b	0	0.00	2	1.40
J total	7	8.86	13	9.09
J1	3	3.80	6	4.20
J2a	1	1.27	6	4.20
J2b	3	3.80	1	0.70
N	1	1.27	0	0.00
Q	5	6.33	4	2.80
R total	32	40.51	80	55.94
R1a	1	1.27	2	1.40
R1b	31	**39.24**	78	**54.55**
T	1	1.27	3	2.10

Bold indicates the most prevalent Y haplogroups. T1D = type 1 diabetes. *In 3 samples the Y haplogroup determination was not obtained due quality control.

The comparative analysis of autosomal ancestry according to Y chromosome categories is described in Table 4. In the T1D group, there were statistically significant differences in African Y and European Y; in both categories, the percentage of European autosomal ancestry was higher than African or Native American (*P* < 0.001). In the control group, patients with African Y showed a higher level of European autosomal ancestry than Native American autosomal ancestry (*P* < 0.001), and patients with European Y showed higher European autosomal ancestry than African or Native American (*P* = 0.003).

**Table 4:** Comparative analysis of autosomal ancestry according to Y chromosome ancestry of the Maranhão state T1D and control groups.

Group	Autosomal ancestry			*P* value
	European	African	Native American	
	Med [IQR]	Med [IQR]	Med [IQR]	
T1D				
Y chromosome				
European	49.4 [36.8–55.8]^a^	24.4 [18.8–31.2]^b^	26.2 [21.2–35.5]^b^	**< 0.001**
African	53.2 [52.3–66.2]^a^	21.1 [15.4–34.0]^b^	15.8 [13.7–25.7]^b^	**< 0.001**
Native American	39.2 [38.4–42.3]	25.1 [22.0–40.4]	31.0 [21.8–35.7]	0.133
Control				
Y chromosome				
European	48.2 [36.3–59.2]^a^	23.0 [16.1–35.3]^b^	23.9 [16.4–32.0]^b^	**0.003**
African	44.8 [30.1–54.5]^a^	29.0 [18.7–48.3]^ab^	25.1 [18.8–31.9]^b^	** 0.001**
Native American	44.1 [30.0–59.2]	21.9 [18.1–31.6]	28.0 [22.6–38.4]	0.129

Data are showed as median and interquartile range [IQR]. Different letters indicate significant differences between Y chromosome categories (ANOVA test port-hoc Bonferroni). T1D = type 1 diabetes. Bold indicates statistical significance (*P* < 0.05).

### HLA analysis and relationship between genetic ancestry markers

The most frequent *HLA-DRB1* alleles (Supplementary Table 2S online) were *03:01* (29.61%), *07:01* (10.2%) and *04:05* (9.21%). The most frequent *HLA-DQA1* alleles (Table 2S) were *05:01* (29.14%), *03:01* (26.82%) and *02:01* (9.93%). The most frequent *HLA-DQB1* alleles (Supplementary Table 2S online) were *03:02* (32.57%), *02:01* (26.32%), and *02:02* (11.84%). For the *DRB1* alleles (Supplementary Table 2S online), higher frequencies of DRB1*04 (30.26%) and DRB1*03 (29.93%) were observed. For *DRB1* genotypes (Supplementary Table 3S online), the most frequent combination was DRB1*03/DRB1*04 (26.32%) followed by DRB1*03/DRB1*03 (8.85%).

For the HLA haplotypes (Table 5), the most frequent ones were DRB1*03:01 ~ DQA1*05:01 ~ DQB1*02:01 (8.22%) and DRB1*03:01 ~ DQA1*03:01 ~ DQB1*02:01 (7.89%). The most frequent HLA genotype was DRB1*03:01 ~ DQA1*05:01 ~ DQB1*02:01 (7.24%) homozygote in the studied sample (Supplementary Table 4S online).

**Table 5:** Distribution of the *HLA-DRB1* ~ *DQA1* ~ *DQB1* haplotypes in patients with T1D in the São Luís, Maranhão State, Brazil (> 1% of frequency).

*HLA-DRB1* ~ *DQA1* ~ *DQB1*	n	%
*03:01* ~ *02:01* ~ *02:01*	8	2.63
***03:01* ~ *03:01* ~ *02:01***	**24**	**7.89**
*03:01* ~ *03:02* ~ *02:01*	10	3.29
***03:01* ~ *05:01* ~ *02:01***	**25**	**8.22**
*04:01* ~ *03:01* ~ *03:02*	8	2.63
*04:01* ~ *05:01* ~ *03:02*	7	2.30
*04:02* ~ *05:01* ~ *03:02*	7	2.30
*04:04* ~ *05:01* ~ *03:02*	10	3.29
*04:05* ~ *03:01* ~ *03:02*	8	2.63
*04:05* ~ *05:01* ~ *03:02*	11	3.62
*07:01* ~ *02:01* ~ *02:02*	7	2.30
*07:01* ~ *05:01* ~ *02:02*	8	2.63

Bold indicates the most prevalent haplotypes. T1D = type 1 diabetes.

Supplementary Fig. 2Sb online shows the distribution of *HLA-DQA1* according to the self-declared color. There was an uneven distribution among the color categories. Among self-reported blacks, the highest frequency was 03:01, while in brown and white it was 05:01.

When compared with the selected controls/BMD, the DRB1*03 and DRB1*04 alleles showed an odds ratio of risk for association with T1D in the evaluated sample. On the other hand, the DRB1*08, DRB1*11, DRB1*13, DRB1*14, and DRB1*15 alleles demonstrated an odds ratio of protection for T1D (Supplementary Table 5S online). The *HLA-DRB1* allele’s OR for risk and protection for T1D in the studied sample is described in Supplementary Table 5S online. The *HLA-DQA1* alleles *03:01, 03:02, 05:03*, and *05:05* showed an OR of risk for association with T1D, while the DQA1*01:01, *01:02*, *01:03*, and *04:01* alleles revealed an OR of protection for T1D (Supplementary Table 5S online). The HLA-DQB1*02:01, *03:02* and *03:19* alleles showed an odds ratio of risk for association with T1D, while the *03:01, 03:03, 04:02, 05:01, 05:03, 06:02,* and *06:03* alleles showed an OR of protection for T1D (Supplementary Table 5S online).

Supplementary Table 6S online shows the distribution of the *HLA- DRB1- DQA1*, and *-DQB1* alleles in patients with T1D according to Y chromosome ancestry. The European Y chromosome showed a higher frequency of DRB1*03 and DRB1*04, while the DRB1*01 and DRB1*16 were the most frequent in the Native American Y (*P* < 0.001). For European Y, the most frequent categories were DQA1*03:01 and *05:01*, while for Native Americans, it was *01:01*. In the European and African Y ancestries, the highest frequencies were observed for DQB1* 02:01 and *03:02*, while for Native American, the highest frequency was *05:01*.

There were significant differences in the distribution of the DRB1*/DRB1* genotype between Y chromosome category (*P* = 0.015). The data showed that DRB1*03 and DRB1*04 homozygote genotypes were detected only in European Y. The frequency of DRB1*03 and DRB1*04 homozygote genotypes and DRB1*03/DRB1*04 heterozygote genotype amounted to 44.9% in European Y patients (Table 6).

**Table 6:** Distribution of the HLA-DRB1*/DRB1* genotypes in patients with T1D according to Y chromosome category.

HLA-DRB1*/DRB1*	EUR (n = 67)		AFR (n = 7)		NAM (n = 5)	
	n	%	n	%	n	%
DRB1*01/DRB1*01	1	1.5	0	0.0	0	0
DRB1*01/DRB1*03	0	0.0	1	14.3	0	0
DRB1*01/DRB1*04	1	1.5	2	28.6	1	20
DRB1*01/DRB1*07	1	1.5	0	0.0	0	0
DRB1*01/DRB1*08	1	1.5	0	0.0	0	0
DRB1*01/DRB1*13	1	1.5	0	0.0	0	0
DRB1*01/DRB1*16	0	0.0	0	0.0	1	20
DRB1*03/DRB1*03	**6**	9.0	0	0.0	0	0
DRB1*03/DRB1*04	**18**	26.9	1	14.3	1	20
DRB1*03/DRB1*07	3	4.5	1	14.3	0	0
DRB1*03/DRB1*08	1	1.5	0	0.0	0	0
DRB1*03/DRB1*11	1	1.5	0	0.0	0	0
DRB1*03/DRB1*16	1	1.5	0	0.0	1	20
DRB1*04/DRB1*04	**6**	9.0	0	0.0	0	0
DRB1*04/DRB1*07	5	7.5	0	0.0	0	0
DRB1*04/DRB1*08	5	7.5	0	0.0	0	0
DRB1*04/DRB1*09	0	0.0	1	14.3	0	0
DRB1*04/DRB1*10	1	1.5	0	0.0	0	0
DRB1*04/DRB1*11	1	1.5	0	0.0	0	0
DRB1*04/DRB1*13	3	4.5	0	0.0	0	0
DRB1*04/DRB1*16	1	1.5	0	0.0	0	0
DRB1*07/DRB1*11	1	1.5	0	0.0	0	0
DRB1*07/DRB1*13	2	3.0	0	0.0	0	0
DRB1*07/DRB1*15	1	1.5	0	0.0	0	0
DRB1*07/DRB1*16	1	1.5	0	0.0	0	0
DRB1*08/DRB1*11	1	1.5	0	0.0	0	0
DRB1*08/DRB1*16	1	1.5	0	0.0	0	0
DRB1*11/DRB1*13	1	1.5	0	0.0	0	0
DRB1*11/DRB1*16	0	0.0	0	0.0	1	20.00
DRB1*13/DRB1*16	0	0.0	1	14.3	0	0
DRB1*14/DRB1*14	1	1.5	0	0.0	0	0
DRB1*15/DRB1*16	1	1.5	0	0.0	0	0

Bold values indicate the most prevalent genotypes.

*EUR* European Y chromosome, *AFR* African Y chromosome, *NAM* Native American Y chromosome. Chi-square test (*P* = 0.015).

The analysis showed that E1b1b and R1b were more frequent in DRB1*03, and R1b was more frequent in DRB1*04 but without statistical significance (*P* = 0.326) (Supplementary Table 7S online). In addition, a network analysis was carried out with all Y-chromosome haplogroups and *HLA-DRB1* alleles in the sample (Supplementary Fig. 3S online).

No significant differences in autosomal ancestry were detected according to the *DRB1* alleles (Supplementary Fig. 4aS online). Significant differences in African ancestry were detected according to *DQA1* (*P* = 0.025), where DQA1*02:02 was more frequent in African ancestry than in the other groups (Supplementary Fig. 4bS online). There were no significant differences in autosomal ancestry for *DQB1* (Supplementary Fig. 4cS online).

## Discussion

Our study showed that individuals with and without T1D from a highly admixed population in Maranhão, a northeastern state of Brazil, have a higher European ancestry. Furthermore, these individuals have a higher percentage of African and Native American ancestry than other Brazilian populations. Concerning the Y chromosome, the most frequent were the ones with a European origin, mainly represented by the haplogroup R1b. The present study also shows that the DRB1*03:01 ~ DQA1*05:01 ~ DQB1*02:01 haplotype was the most frequent in individuals with T1D, being the most prevalent risk alleles following DRB1*03 and DRB1*04. These data are like other studies performed in Brazil, as well in other countries. Moreover, an important finding was the relationship between European Y chromosome with DRB1*03 and DRB1*04 homozygous and DRB1*03/DRB1*04 heterozygous genotypes in the T1D individuals. The above-mentioned data emphasized that although being an admixed population, Brazilian people still have a great influence from European autosomal and Y chromosome ancestry. Besides, even though the Brazilian population has European, Native American, and African as ancestries’ roots, the process of miscegenation could be quite different among the different regions, highlighting the importance to carry out studies in different states of a continental country, like Brazil.

The population from the state of Maranhão, located in the Northeast region of Brazil, is also composed by the miscegenation between European, African, and Native American populations^10,25^. This fact was noted in our study through the analysis of genomic ancestry. However, the percentages of miscegenation between these ethnicities are quite different in each Brazilian region, depending on the specific colonization process and the geographical area^10,16,24^. Through our analyses we found that, as in all Brazilian regions, European ancestry was the largest contributor, but in our population, it approached 50% in both groups (T1D and controls), differing from the weighted average of 68.1% found in the Brazilian population in a systematic review study conducted in 2019^24^. We also obtained a similar percentage between African and Native American ancestry (around 25% each), which again differs from the Brazilian average of 19.6% African and 11.6% Native American^24^. A possible explanation for this difference is due to the identification of three asymmetric mating models in Maranhão. In almost all Brazilian populations, an asymmetric mating pattern usually occurs, preferably between European men and Native American or African women^10^. However, in Afro-descendant communities in Maranhão and the Amazon, another pattern of asymmetric mating was observed, occurring between African men and Native American women^22^. Still, in Maranhão, the Guajajaras American Native also maintained contact with the Brazilian population, which is already a mixed race and, with African slaves, Guajajaras men mating with African or mixed women being more common^23^.

When performing the analysis of ancestry-specific principal components analysis with the panel of the HGDP-CEPH^31^, we observed that the samples from Maranhão are grouped closer to the Europeans. When compared to a database of a healthy Brazilian population from all geographic regions of Brazil^10^, they are closer to Native Americans. These findings corroborate the aforementioned facts.

Historically, the highest incidence of T1D occurs in whites of European ancestry^2,36^. In our study, we observed a predominance of similar European autosomal ancestry in the T1D and control groups (47.3 and 48.5, respectively), which was different from that found in a large Brazilian analysis carried out by Gomes et al., where there was a higher percentage of European ancestry in the T1D individuals than in the control group (67.8 and 56.3, respectively)^17^, which was in agreement with another study (77 and 71; respectively) also in Brazil, conducted only in the state of São Paulo^37^. This finding in our sample can be explained by the increasing incidence of T1D in ethnic minorities, as has been observed in the USA^36^. However, despite the great miscegenation in our population, all T1D individuals had at least 35% European ancestry.

In the colonization process of Maranhão, there were some differences with the rest of Brazil, mainly in the origin of the Europeans involved^18–20^. In 1535, the Portuguese arrived in the lands of Maranhão and met the Native American people^18^. In 1607, the French people landed on the island, and in 1615, they handed over the São Luís fortress to the Portuguese^18,19^, remaining in their domain until 1641, when it was invaded by the Dutch, expelled 2 years later^18,20^. The first historical records of the entry of slaves in Maranhão dates from around 1655 and ends in 1831. It is estimated that about 187,000 African slaves joined and that in 1822, they corresponded to 50% of the population of Maranhão. These Africans were mainly from Guinea-Bissau, Togo, Benin, Nigeria, and Angola and to a lesser extent Senegal, Gambia, Guinea, Alto-Volta, Ghana, Congo, and the archipelagos of Cape Verde and São Tomé and Príncipe^21^.

Despite the presence of these three ethnic groups in Maranhão, the male contribution to the miscegenation process was predominantly European in all regions of Brazil^10,29^, which is confirmed in the study of our population with and without T1D, which had a predominance of the European Y chromosome, with the R1b haplogroup being the most frequent.

The R1b has a high frequency in western Europe, including in the original countries of the people of Maranhão, with percentages reaching approximately 57 in Portugal^38^ and in the Netherlands^39^, and 68.7 in France^40^. In the T1D group, it was 39.24% and 54.55% in the control group. To clarify the different sub-haplogroups of R1b, with the possibility of being more specific in their phylogeography and specific origin in each of these countries, we should use Y-SNPs^29^, which was not possible in the present study.

The second most frequent haplogroup in the T1D group was E1b1b (17.72%) and in the control group, it was E1b1a (9.79%). The haplogroup E is seen in Africa, Europe, and the Middle East and includes several subhaplogroups with different distributions on these continents. Some sublineages are from sub-Saharan Africa, such as E1b1a. Other E1b1b subhaplogroups have a similar frequency in Africa and Europe (E1b1b-M78), with a high prevalence in North Africa, and in the Iberian Peninsula (E1b1b- M81) and in West Asia and Europe (E1b1b-M123)^41^. We believe that this higher frequency of E1b1b is due to the Portuguese influence in our population, since in a study conducted by Martiniano et al., E1b1b had the third-highest frequency (12.0%) in the studied Portuguese population^38^.

The haplogroup Q exhibits Asian descendants and has established itself in the Americas^42^, being almost restricted to the Native American population and currently uncommon in the admixed Brazilian population. In a study carried out with the population of the different regions of Brazil, only 3.1% of the Y chromosomes belonging to the Q were found^29^. In our sample, we obtained it in the T1D group (6.33%) and in the control group (2.80%). This greater contribution of Native Americans in our study may be due to the diversity of asymmetric miscegenation models found in Maranhão, as detailed above.

The analysis of autosomal ancestry and Y chromosome ancestry in our population detected an important European influence. Concerning these latter facts, we have performed a hypothesis relating both ancestries with the most important worldwide genetic susceptibility marker to diabetes type 1: the HLA system.

The DRB1*03 and DRB1*04 alleles are the most frequent risk alleles in individuals with T1D^43^, especially in European populations^1^. As expected, our results showed that when comparing our T1D group, the CRsr and regional REDOME controls, the DRB1*03 and DRB1*04 alleles showed an odds ratio of risk for association with T1D, with DRB1*04 (30.26%), DRB1*03 (29.93%) and the DR3/DR4 heterozygous genotype (26.32%) as the most frequents. In a large analysis of the T1D population in all Brazilian regions, a result similar to ours was found^44^. It is reported that approximately 30% of individuals with T1D have DR3/DR4 in heterozygosity^6,45^, corroborating our findings.

In Europeans, the highest risk for the disease is associated with the DRB1*04:01/02/04/05 ~ DQA1*03 ~ DQB1*03:02 (DR4-DQ8) haplotypes and with DRB1*03:01 ~ DQA1*05:01 ~ DQB1*02:01 (DR3-DQ2)^1,6,7^. In our study, the most frequent haplotype was DR3-DQ2 (5.63%), and the most frequent HLA genotype was DRB1*03:01 ~ DQA1*05:01 ~ DQB1*02:01 (DR3-DQ2) homozygote (7.24%). We still found that the most frequent *DQA1* alleles were *05:01* (29.14%), and *03:01* (26.82%), and the most frequent *DQB1* alleles were *03:02* (32.57%), *02:01* (26.32%), compatible with the fact that these are the most frequent *DQA1* and *DQB1* alleles associated with T1D^4^. In a study performed by Santos et al., it was observed that in Brazilian individuals with T1D, the same haplotype, DRB1*03:01 ~ DQA1*05:01 ~ DQB1*02:01 (DR3-DQ2), was the most frequently found in this subpopulation of individuals^44^.

When we analyzed the relationship of the DRB1*03 and DRB1*04 alleles with the CRsr, we noticed its greater frequency in self-reported white and brown individuals. Some studies have suggested that there is no good correlation between self-reported color and ancestry in Brazilian individuals^12,46^, but in our analysis, we found that in both groups (T1D and Control), European ancestry was predominant among the self-reported white and brown people (P < 0.05). In another Brazilian survey, HLA-DRB1*03 and DRB1*04 were also more prevalent in the self-reported white people^44^.

The Y chromosome polymorphisms allow discrimination between individuals within a population and shorter biogeographical inference of their paternal ancestry^15,47^. To the best of our knowledge, this is the first study relating the ancestry of the paternal lineage (Y chromosome) with the genotyping of HLA class II in T1D individuals.

We observed that when the Y chromosome was Native American (haplogroup Q), DRB1*01, DRB1*16, DQA1*01:01, and DQB1*05:01 were the most frequent, differing from the other groups. In Native American populations supposedly without non-Amerindian gene flow, only five *HLA-DRB1* allele strains are commonly observed (DRB1*04, DRB1*08, DRB1*09, DRB1*14, and DRB1*16). Arnaiz-Villena et al. described that HLA genes do not confer susceptibility to T1D in Native Americans unless if these people are mixed with Europeans. According to this same author, only one case of T1D in an Amerindian individual without a European mixture was reported^48^. We consider that the low casuistry of Native American Y chromosomes and the presence of a significant percentage of European ancestry in our sample do not allow us to infer the influence of the *HLA-DRB1, DQA1,* and *DQB1* alleles according to the Native American Y chromosome ancestry.

When we analyzed the European Y, we found that the most frequent alleles were DRB1*03 and DRB1*04; DQA1*03:01 and 05:01; and DQB1*02:01 and *03:02*. The DRB1*03 or DRB1*04 homozygous genotype detected only in European Y and the high frequency of European Y in the DRB1*03/DRB1*04 heterozygous genotype suggest the association of these alleles, known to be the most frequent in the European population^1^, with a European ancestry of the Y chromosome in our sample. However, these results should be interpreted with caution, since African and Native American Y sample sizes were small in the present study. Thus, further studies should be conducted with large admixed sample sizes to clarify those possible associations.

Our study has some limitations. The color/race of the family members was informed by the participants, since the individual's perception of his/her color/race may be different from a third family member; moreover, we can not exclude that social factors may also affect CRsr. However, we did not have access to the self-reported information of many family members, because they are geographically distant or deceased, especially grandparents and great-grandparents. Also, when performing the analysis of ancestry-specific principal component analysis of the autosomal ancestry of the panel of 46 INDEL markers, we did not obtain a similar database from the Portuguese population, which is not included in the HGDP-CEPH^31^ for comparison, being a limiting factor, as it is one of the most important origins of the Brazilian population. We also consider a limitation of the fact that we used a second control/BMD group from the REDOME database; nonetheless, this was pair-matched by region and CRsr. Although information of Y haplogroup was not available in the REDOME, we may suggest, due to the large interquartile range of autosomal ancestry in selected individuals from REDOME CRsr brown and white, that they share the HLA alleles of similar Y haplogroups.

The strength of our study is the more specific assessment of the ancestry of individuals with T1D in a highly miscegenated population, using genomic and Y chromosome markers. We consider that the use of healthy individuals in control groups is also an important factor in our analysis. Besides, we believe it is the first study correlating patrilineal ancestry with HLA class II analysis in T1D individuals.

In conclusion, our study demonstrated that individuals with and without T1D in Maranhão have European origin as their largest component, and African and Native American percentage higher than other Brazilian populations. The European patrilineal origin was evidenced by the higher frequency of the R1b haplogroup. The predominance of the HLA-DRB1*03 and DRB1*04 alleles conferring greater risk in our population and being more frequently related to the ancestry of the European Y chromosome, suggests that in our population, the risk of T1D may have been transmitted by European ancestors during our miscegenation process. However, the Y sample sizes of Africans and Native Americans were small, and further research should be conducted with large mixed sample sizes to clarify this possible association. To fill the knowledge gap of the ancestry and genetic origin of T1D in an admixed population like the Brazilian one, further studies with other ancestry markers, in conjunction with the analysis of HLA class II, in T1D individuals and controls need to be addressed.

## Supplementary Information

Supplementary Information.
