# Supplementary material for: Genetic ancestry inferred from autosomal and Y chromosome markers and HLA genotypes in Type 1 Diabetes from an admixed Brazilian population
Source: Sci Rep. 2021 Jul 8;11:14157. doi: 10.1038/s41598-021-93691-x (PMC8266844; doi:10.1038/s41598-021-93691-x)
Supplement: Supplementary file 1 — Supplementary Information. [file 41598_2021_93691_MOESM1_ESM.docx]

**Genetic ancestry inferred from autosomal and Y chromosome markers and HLA genotypes in Type 1 Diabetes from an admixed Brazilian population.**

Rossana Santiago de Sousa Azulay^1,2^*, Luís Cristóvão Porto^3^, Dayse Aparecida Silva^4^, Maria da Gloria Tavares^1,2^, Roberta Maria Duailibe Ferreira Reis^2^, Gilvan Cortês Nascimento^1,2^, Sabrina da Silva Pereira Damianse^1,2^, Viviane Chaves de Carvalho Rocha^1,2^, Marcelo Magalhães^2,5^, Vandilson Rodrigues^2^, Paulo Ricardo Vilas Boas Carvalho^3^, Manuel dos Santos Faria^1,2,5^, Marília Brito Gomes^6^

^1^Service of Endocrinology, University Hospital of the Federal University of Maranhão (HUUFMA/EBSERH), São Luís, Brazil

^2^Research Group in Clinical and Molecular Endocrinology and Metabology (ENDOCLIM), São Luís, Brazil

^3^Histocompatibility and Cryopreservation Laboratory (HLA), Rio de Janeiro State University (UERJ), Rio de Janeiro, Rio de Janeiro, Brazil

^4^DNA Diagnostic Laboratory (LDD), Rio de Janeiro State University (UERJ), Rio de Janeiro, Rio de Janeiro, Brazil

^5^Clinical Research Center of the University Hospital of the Federal University of Maranhão (CEPEC – HUUFMA), São Luís, Brazil

^6^Diabetes Unit, State University of Rio de Janeiro (UERJ), Rio de Janeiro, Brazil

* Corresponding author at: Service of Endocrinology, University Hospital, Federal University of Maranhão (HUUFMA), Rua Barão de Itapary, 227, Centro, 65020-070, São Luís, Maranhão, Brazil. *E-mail address*: rossanaendocrino@gmail.com

**Table 1S. Color/race of family members informed of the Maranhão state T1D and control participants.**

| **Variable** | **T1D**  **(n=152)** | **Control**  **(n=286)** | ***P* value** |
| --- | --- | --- | --- |
|  | **n (%)** | **n (%)** |  |
| Reported color-race |  |  |  |
| Father |  |  | 0.086 |
| White | 42 (27.6) | 84 (29.4) |  |
| Black | 18 (11.8) | 57 (19.9) |  |
| Brown* | 85 (55.9) | 135 (47.2) |  |
| Asian* | 0 (0.0) | 1 (0.3) |  |
| Indigenous | 1 (0.7) | 5 (1.8) |  |
| Unknown | 6 (3.9) | 4 (1.4) |  |
| **Mother** |  |  | **0.010** |
| White | 44 (28.9) | 98 (34.3) |  |
| Black | 13 (8.6) | 45 (15.7) |  |
| Brown* | 94 (61.8) | 131 (45.8) |  |
| Asian* | 0 (0.0) | 4 (1.4) |  |
| Indigenous | 0 (0.0) | 6 (2.1) |  |
| Unknown | 1 (0.7) | 2 (0.7) |  |
| Maternal grandfather |  |  | 0.183 |
| White | 45 (29.6) | 77 (27.0) |  |
| Black | 19 (12.5) | 60 (21.1) |  |
| Brown* | 70 (46.1) | 105 (36.8) |  |
| Asian* | 0 (0.0) | 1 (0.3) |  |
| Indigenous | 1 (0.7) | 3 (1.1) |  |
| Unknown | 17 (11.2) | 39 (13.7) |  |
| Maternal grandmother |  |  | 0.609 |
| White | 61 (40.1) | 108 (37.8) |  |
| Black | 15 (9.9) | 37 (12.9) |  |
| Brown* | 60 (39.5) | 107 (37.4) |  |
| Asian* | 0 (0.0) | 2 (0.7) |  |
| Indigenous | 2 (1.3) | 9 (3.2) |  |
| Unknown | 14 (9.2) | 23 (8.0) |  |
| Paternal grandfather |  |  | 0.824 |
| White | 43 (28.3) | 80 (28.0) |  |
| Black | 21 (13.8) | 51 (17.8) |  |
| Brown* | 48 (31.6) | 87 (30.4) |  |
| Asian* | 0 (0.0) | 1 (0.3) |  |
| Indigenous | 1 (0.7) | 3 (1.1) |  |
| Unknown | 39 (25.7) | 64 (22.4) |  |
| Paternal grandmother |  |  | 0.192 |
| White | 55 (36.2) | 94 (32.9) |  |
| Black | 13 (8.6) | 44 (15.4) |  |
| Brown* | 54 (35.5) | 91 (31.8) |  |
| Asian* | 0 (0.0) | 2 (0.7) |  |
| Indigenous | 1 (0.7) | 7 (2.4) |  |
| Unknown | 29 (19.1) | 48 (16.8) |  |
| Data are presented as number (percentage). T1D = type 1 diabetes. Bold indicates statistical significance (*P* < .05). | | | |

**Figure 1S. Box-plot representing autosomal ancestry within self-reported color-race groups in patients with T1D (a) and control group (b).** EUR = European. AFR = African. NAM = Native American.

**Table 2S. Most frequent (> 5% of frequency) *HLA-DRB1*, *-DQA1* and *-DQB1*alleles in patients with T1D in the São Luís, Maranhão State, Brazil.**

| **HLA allele** | **n** | **%** |
| --- | --- | --- |
| ***DRB1**** |  |  |
| *01* | 22 | 7.24% |
| ***03*** | **91** | **29.93%** |
| ***03:01*** | **90** | **29.61%** |
| ***04*** | **92** | **30.26%** |
| *04:01* | 21 | 6.91% |
| *04:04* | 18 | 5.92% |
| ***04:05*** | **28** | **9.21%** |
| ***07:01*** | **31** | **10.20%** |
| ***DQA1**** |  |  |
| *01:01* | 27 | 8.94% |
| *01:02* | 19 | 6.29% |
| ***03:01*** | **81** | **26.82%** |
| *03:02* | 21 | 6.95% |
| ***05:01*** | **88** | **29.14%** |
| *05:05* | 18 | 5.96% |
| ***DQB1**** |  |  |
| ***02:01*** | **80** | **26.32%** |
| ***02:02*** | **36** | **11.84%** |
| *03:01* | 19 | 6.25% |
| ***03:02*** | **99** | **32.57%** |
| *05:01* | 29 | 9.54% |
| Bold indicates the most prevalent HLA alleles. T1D = type 1 diabetes. | | |

**Table 3S. *HLA-DRB1/DRB1* genotypes distribution in patients with T1D in the São Luís, Maranhão State, Brazil (> 1% of frequency).**

| ***HLA-DRB1/DRB1 genotype*** | **n** | **%** |
| --- | --- | --- |
| *DRB1*01/DRB1*10* | 2 | 1.32% |
| *DRB1*01/DRB1*13* | 2 | 1.32% |
| *DRB1*01/DRB1*03* | 5 | 3.29% |
| *DRB1*01/DRB1*04* | 6 | 3.95% |
| ***DRB1*03/DRB1*03*** | **13** | **8.55%** |
| ***DRB1*03/DRB1*04*** | **40** | **26.32%** |
| ***DRB1*03/DRB1*07*** | **8** | **5.27%** |
| *DRB1*03/DRB1*09* | 2 | 1.32% |
| *DRB1*03/DRB1*11* | 2 | 1.32% |
| *DRB1*03/DRB1*13* | 4 | 2.63% |
| *DRB1*03/DRB1*16* | 2 | 1.32% |
| ***DRB1*04/DRB1*04*** | **9** | **5.92%** |
| ***DRB1*04/DRB1*07*** | **9** | **5.92%** |
| *DRB1*04/DRB1*08* | 7 | 4.61% |
| *DRB1*04/DRB1*09* | 3 | 1.97% |
| *DRB1*04/DRB1*10* | 2 | 1.32% |
| *DRB1*04/DRB1*11* | 2 | 1.32% |
| *DRB1*04/DRB1*13* | 4 | 2.63% |
| *DRB1*07/DRB1*07* | 2 | 1.32% |
| *DRB1*07/DRB1*13* | 2 | 1.32% |
| *DRB1*07/DRB1*15* | 2 | 1.32% |
| Bold indicates the most prevalent HLA-DRB1 genotypes. T1D = type 1 diabetes. | | |

**Table 4S. Frequencies of HLA genotypes in patients with T1D in the São Luís, Maranhão State, Brazil (> 1% of frequency).**

| **Genotype**  ***DRB1~DQA1~DQB1 DRB1~DQA1~DQB1*** | **n** | **%** |
| --- | --- | --- |
| *01:01~01:01~02:01 03:01~05:01~05:01* | 2 | 1.32% |
| *01:01~01:01~03:02 04:05~03:01~05:01* | 3 | 1.97% |
| *01:02~01:01~02:01 03:01~05:01~05:01* | 3 | 1.97% |
| *01:02~01:01~05:01 10:01~01:01~05:01* | 2 | 1.32% |
| ***03:01~02:01~02:01 07:01~05:01~02:02*** | **8** | **5.26%** |
| *03:01~03:01~02:01 04:01~05:01~03:02* | 6 | 3.95% |
| ***03:01~03:01~02:01 04:02~05:01~03:02*** | **7** | **4.61%** |
| ***03:01~03:01~02:01 04:04~05:01~03:02*** | **7** | **4.61%** |
| *03:01~03:01~03:02 04:04~05:01~03:02* | 3 | 1.97% |
| ***03:01~03:02~02:01 04:05~05:01~03:02*** | **9** | **5.92%** |
| *03:01~03:02~03:02 04:05~05:01~03:02* | 2 | 1.32% |
| ***03:01~05:01~02:01 03:01~05:01~02:01*** | **11** | **7.24%** |
| *04:01~03:01~03:02 04:05~03:01~03:02* | 3 | 1.97% |
| *04:01~03:01~03:02 08:07~04:01~04:02* | 3 | 1.97% |
| *04:04~02:01~02:02 07:01~03:01~03:02* | 2 | 1.32% |
| *04:05~02:01~02:02 07:01~03:01~03:02* | 3 | 1.97% |
| *04:07~01:01~03:02 10:01~03:01~05:01* | 2 | 1.32% |
| *07:01~01:02~02:02 15:03~02:01~06:02* | 2 | 1.32% |
| *07:01~02:01~02:02 09:01~03:01~02:02* | 2 | 1.32% |
| Bold indicates the most prevalent categories. | | |

**Figure 2S. Distribution of the *HLA-*DR*B1* (a) and *HLA-DQA1* (b)alleles in patients with T1D in the São Luís, Maranhão State according to self-reported color/race.**

**Table 5S. Allelic frequency distribution of the *HLA-DRB1* in individuals with type 1 diabetes and control/BMD.**

| **HLA alleles** | **T1D** | **Control/REDOME** | **OR [95% CI]** | ***P* value** |
| --- | --- | --- | --- | --- |
|  | **n (%)** | **n (%)** |  |  |
| ***DRB1**** |  |  |  |  |
| *01* | 22 (7.24) | 117 (9.44) | 0.74 [0.46–1.20] | 0.230 |
| *03* | **91 (29.93)** | 94 (7.58) | **5.20 [3.77–7.19]** | **<0.001*** |
| *04* | **92 (30.26)** | **165 (13.31)** | **2.82 [2.10–3.79]** | **<0.001*** |
| *07* | 31 (10.2) | **167 (13.47)** | 0.72 [0.48–1.09] | 0.126 |
| *08* | 13 (4.28) | 98 (7.90) | **0.52 [0.28–0.94]** | **0.028*** |
| *09* | 8 (2.63) | 32 (2.58) | 1.02 [0.46–2.23] | 1.000 |
| *10* | 4 (1.32) | 27 (2.18) | 0.59 [0.20–1.72] | 0.337 |
| *11* | 9 (2.96) | 132 (10.65) | **0.25 [0.12–0.50]** | **<0.001*** |
| *12* | 1 (0.33) | 8 (0.65) | 0.50 [0.06–4.07] | 1.000 |
| *13* | 14 (4.61) | **173 (13.95)** | **0.29 [0.17–0.52]** | **<0.001*** |
| *14* | 2 (0.66) | 56 (4.52) | **0.14 [0.03–0.57]** | **0.001*** |
| *15* | 5 (1.64) | 101 (8.15) | **0.18 [0.07–0.46]** | **<0.001*** |
| *16* | 12 (3.95) | 70 (5.65) | 0.68 [0.36–1.28] | 0.236 |
| ***DQA1**** |  |  |  |  |
| *01:01* | 27 (8.94) | 169 (13.63) | **0.61 [0.40-0.94]** | **0.024*** |
| *01:02* | 19 (6.29) | **188 (15.16)** | **0.37 [0.22-0.60]** | **<0.001*** |
| *01:03* | 5 (1.66) | 82 (6.61) | **0.23 [0.09-0.58]** | **<0.001*** |
| ***02:01*** | **30 (9.93)** | 169 (13.63) | 0.69 [0.46-1.04] | 0.079 |
| ***03:01*** | **81 (26.82)** | **209 (16.85)** | **1.79 [1.33-2.40]** | **<0.001*** |
| *03:02* | 21 (6.95) | 0 (0) | - | **<0.001*** |
| *04:01* | 10 (3.31) | 98 (7.90) | **0.39 [0.20-0.76]** | **0.004*** |
| *04:02* | 0 (0) | 1 (0.08) | - | 1.000 |
| ***05:01*** | **88 (29.14)** | **322 (25.97)** | 1.16 [0.87-1.53] | 0.292 |
| *05:03* | 2 (0.66) | 0 (0) | - | **0.038*** |
| *05:05* | 18 (5.96) | 0 (0) | - | **<0.001*** |
| *05:10* | 0 (0) | 1 (0.8) | - | 1.000 |
| *06:01* | 1 (0.33) | 1 (0.8) | 4.08 [0.25-65.65] | 0.355 |
| ***DQB1**** |  | |  |  |
| ***02:01*** | **80 (26.32)** | 83 (6.69) | 4.97 [3.54-6.98] | **<0.001*** |
| ***02:02*** | **36 (11.84)** | **157 (12.66)** | 0.92 [0.62-1.36] | 0.698 |
| *03:01* | 19 (6.25) | **255 (20.56)** | 0.25 [0.15-0.41] | **<0.001*** |
| ***03:02*** | **99 (32.57)** | 134 (10.81) | 3.98 [2.95-5.37] | **<0.001*** |
| *03:03* | 5 (1.64) | 57 (4.60) | 0.34 [0.13-0.87] | **0.018*** |
| *03:04* | 0 (0) | 2 (0.16) | - | 1.000 |
| *03:19* | 3 (0.99) | 0 (0) | - | **0.007*** |
| *04:01* | 0 (0) | 1 (0.08) | - | 1.000 |
| *04:02* | 12 (3.95) | 112 (9.03) | 0.41 [0.22-0.76] | **0.003*** |
| *05:01* | 29 (9.54) | **161 (12.98)** | 0.70 [0.46-1.07] | **0.001*** |
| *05:02* | 3 (0.99) | 17 (1.37) | 0.71 [0.20-2.46] | 0.780 |
| *05:03* | 0 (0) | 21 (1.69) | - | **0.022*** |
| *06:01* | 0 (0) | 4 (0.32) | - | 1.000 |
| *06:02* | 6 (1.97) | 109 (8.79) | 0.20 [0.09-0.47] | **<0.001*** |
| *06:03* | 5 (1.64) | 78 (6.29) | 0.24 [0.10-0.62] | **0.001*** |
| *06:04* | 6 (1.97) | 29 (2.34) | 0.84 [0.34-2.04] | 0.698 |
| *06:09* | 1 (0.33) | 17 (1.37) | 0.23 [0.03-1.79] | 0.227 |
| *06:11* | 0 (0) | 2 (0.16) | - | 1.000 |
| *06:38* | 0 (0) | 1 (0.08) | - | 1.000 |
| OR = Odds ratio. 95% CI = 95% confidence interval. * Statistically significant difference (P < .05). Control/REDOME = Samples from REDOME database. | | | | |

**Table 6S. Allelic frequency distribution of the HLA-*DRB1*, *-DQA1* and *-DQB1* in individuals with T1D according to Y chromosome ancestry.**

| **HLA Alleles** | **Y chromosome ancestry** | | | ***P* value** | | |
| --- | --- | --- | --- | --- | --- | --- |
|  | **EUR**  **(n = 134)** | **AFR**  **(n = 14)** | **NAM**  **(n = 10)** | **EUR *versus* AFR** | **EUR *versus* NAM** | **AFR *versus* NAM** |
|  | **n (%)** | **n (%)** | **n (%)** |  |  |  |
| ***DRB1**** |  |  |  | 0.785 | <0.001* | 0.525 |
| *01* | 6 (4.5) | 2 (14.4) | 3 (30.0) |  |  |  |
| *03* | 36 (26.9) | 4 (28.6) | 2 (20.0) |  |  |  |
| *04* | 47 (35.1) | 4 (28.6) | 1 (10.0) |  |  |  |
| *07* | 14 (10.4) | 1 (7.1) | 0 (0) |  |  |  |
| *08* | 9 (6.7) | 0 (0) | 0 (0) |  |  |  |
| *13* | 7 (5.2) | 1 (7.1) | 0 (0) |  |  |  |
| *16* | 5 (3.7) | 1 (7.1) | 3 (30.0) |  |  |  |
| Other | 10 (7.5) | 1 (7.1) | 1 (10.0) |  |  |  |
| ***DQA1**** |  |  |  | 0.277 | 0.108 | 0.499 |
| *01:01* | 8 (6.1) | 2 (14.3) | 3 (30.0) |  |  |  |
| *01:02* | 10 (7.6) | 1 (7.1) | 2 (20.0) |  |  |  |
| *01:03* | 3 (2.3) | 0 (0) | 0 (0) |  |  |  |
| *02:01* | 12 (9.1) | 1 (7.1) | 0 (0) |  |  |  |
| *03:01* | 42 (31.8) | 2 (14.3) | 1 (10.0) |  |  |  |
| *03:02* | 5 (3.8) | 3 (21.4) | 0 (0) |  |  |  |
| *04:01* | 6 (4.5) | 0 (0) | 0 (0) |  |  |  |
| *05:01* | 35 (26.5) | 4 (28.6) | 2 (20.0) |  |  |  |
| *05:03* | 2 (1.5) | 0 (0) | 0 (0.0) |  |  |  |
| *05:05* | 9 (6.8) | 1 (7.1) | 2 (20.0) |  |  |  |
| ***DQB1**** |  |  |  | 0.963 | 0.086 | 0.374 |
| *02:01* | 31 (23.1) | 4 (28.6) | 2 (20.0) |  |  |  |
| *02:02* | 12 (9.0) | 2 (14.3) | 0 (0) |  |  |  |
| *03:01* | 12 (9.0) | 1 (7.1) | 2 (20.0) |  |  |  |
| *03:02* | 49 (36.6) | 4 (28.6) | 1 (10.0) |  |  |  |
| *03:03* | 1 (0.7) | 0 (0) | 0 (0) |  |  |  |
| *03:19* | 2 (1.5) | 0 (0) | 0 (0) |  |  |  |
| *04:02* | 7 (5.2) | 0 (0) | 0 (0) |  |  |  |
| *05:01* | 9 (6.7) | 2 (14.3) | 3 (30.0) |  |  |  |
| *05:02* | 2 (1.5) | 0 (0) | 1 (10.0) |  |  |  |
| *06:02* | 3 (2.2) | 0 (0) | 0 (0) |  |  |  |
| *06:03* | 2 (1.5) | 0 (0) | 1 (10.0) |  |  |  |
| *06:04* | 4 (3.0) | 1 (7.1) | 0 (0) |  |  |  |
| EUR = European Y chromosome. AFR = African Y chromosome. NAM = Native American Y chromosome. Other = *DRB1* 09, 10, 11, 14, 15*. * Statistically significant difference (*P* < 0.05). | | | | | | |

**Table 7S. Distribution of the *DRB1** alleles in individuals with T1D according to Y chromosome haplogroup.**

| **HLA** | **Y chromosome haplogroup** | | | | | | | | | | | | | |
| --- | --- | --- | --- | --- | --- | --- | --- | --- | --- | --- | --- | --- | --- | --- |
|  | **E1b1a** | | **E1b1b** | | **G2a** | | **I2a** | | **Q** | | **R1b** | | **Other** | |
|  | **n** | **%** | **n** | **%** | **n** | **%** | **n** | **%** | **n** | **%** | **n** | **%** | **n** | **%** |
| *DRB1*01* | 2 | 18.2 | 0 | 0 | 0 | 0 | 1 | 9.1 | **3** | **27.3** | 2 | 18.2 | **3** | **27.3** |
| *DRB1*03* | 4 | 9.5 | **10** | **23.8** | 4 | 9.5 | 6 | 14.3 | 2 | 4.8 | **10** | **23.8** | 6 | 14.3 |
| *DRB1*04* | 3 | 5.8 | 9 | 17.3 | 3 | 5.8 | 1 | 1.9 | 1 | 1.9 | **26** | **50.0** | 9 | 17.3 |
| *DRB1*07* | 1 | 6.7 | 2 | 13.3 | 1 | 6.7 | 1 | 6.7 | 0 | 0 | **9** | **60.0** | 1 | 6.7 |
| *DRB1*08* | 0 | 0 | 1 | 11.1 | 1 | 11.1 | 1 | 11.1 | 0 | 0 | **3** | **33.3** | **3** | **33.3** |
| *DRB1*09* | 0 | 0 | 0 | 0 | 0 | 0 | 0 | 0 | 0 | 0 | 0 | 0 | **1** | **100** |
| *DRB1*10* | 0 | 0 | **1** | **100** | 0 | 0 | 0 | 0 | 0 | 0 | 0 | 0 | 0 | 0 |
| *DRB1*11* | 0 | 0 | 1 | 16.7 | 1 | 16.7 | 0 | 0 | 1 | 16.7 | 1 | 16.7 | **2** | **33.2** |
| *DRB1*13* | 1 | 12.5 | 2 | 25.0 | 0 | 0 | 0 | 0 | 0 | 0 | **4** | **50.0** | 1 | 12.5 |
| *DRB1*14* | 0 | 0 | 0 | 0 | 0 | 0 | 0 | 0 | 0 | 0 | **2** | **100** | 0 | 0 |
| *DRB1*15* | 0 | 0 | **1** | **50.0** | 0 | 0 | 0 | 0 | 0 | 0 | **1** | **50.0** | 0 | 0 |
| *DRB1*16* | 1 | 11.1 | 1 | 11.1 | 0 | 0 | 0 | 0 | 3 | 33.3 | **4** | **44.4** | 0 | 0 |
| Other = Frequency less than 5 (T, E1a, I1, I2b, J1, J2a, J2b, N, R1a). Bold indicates Y haplogroup most frequent according to *HLA-DRB1*. Chi-square test (*P*=0.326) | | | | | | | | | | | | | | |


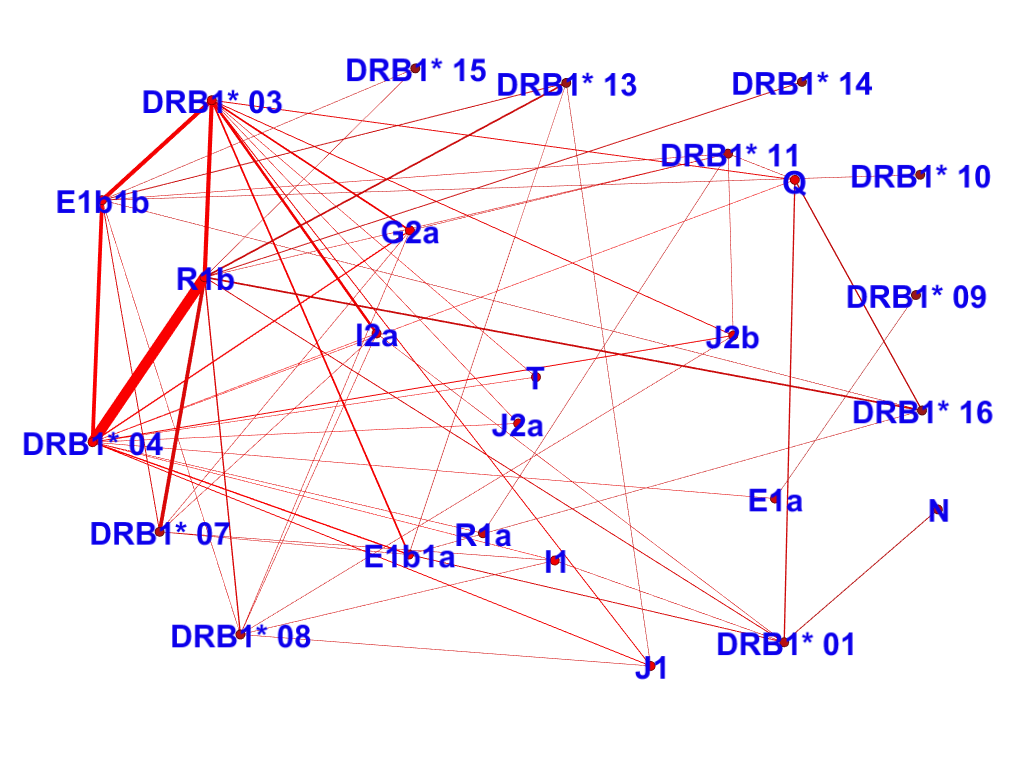


**Figure 3S. Network analysis between Y-chromosome haplogroups and HLA-DRB1* alleles. Line thickness between nodes indicates frequency of relationships between Y-chromosome haplogroup and HLA-DRB1* allele.**

**
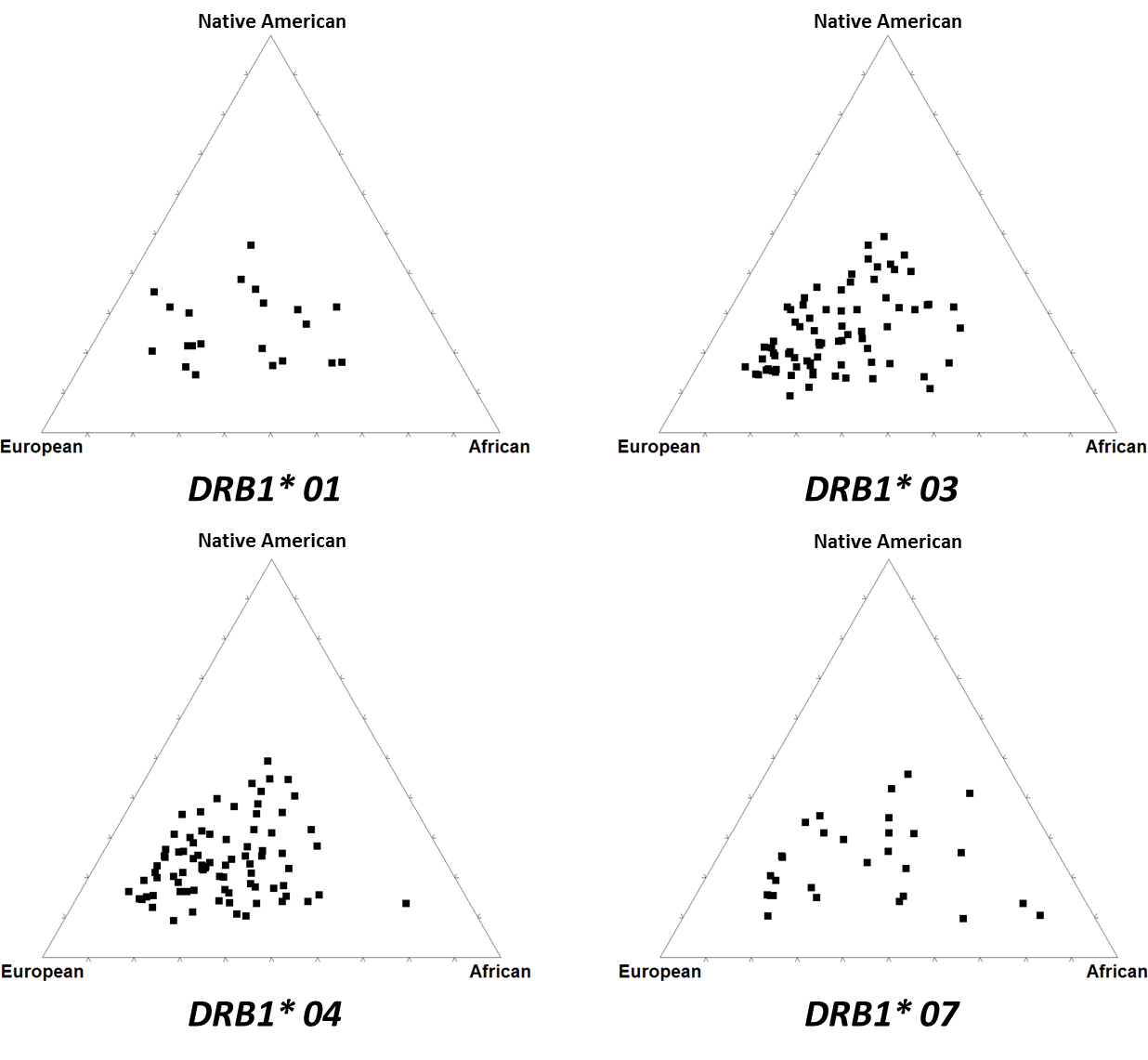
**

**Figure 4aS. Triangle plot of autosomal ancestry according to *DRB1* alelles.**

African (p = 0.078), Native American (p = 0.692), European (p = 0.215)

**
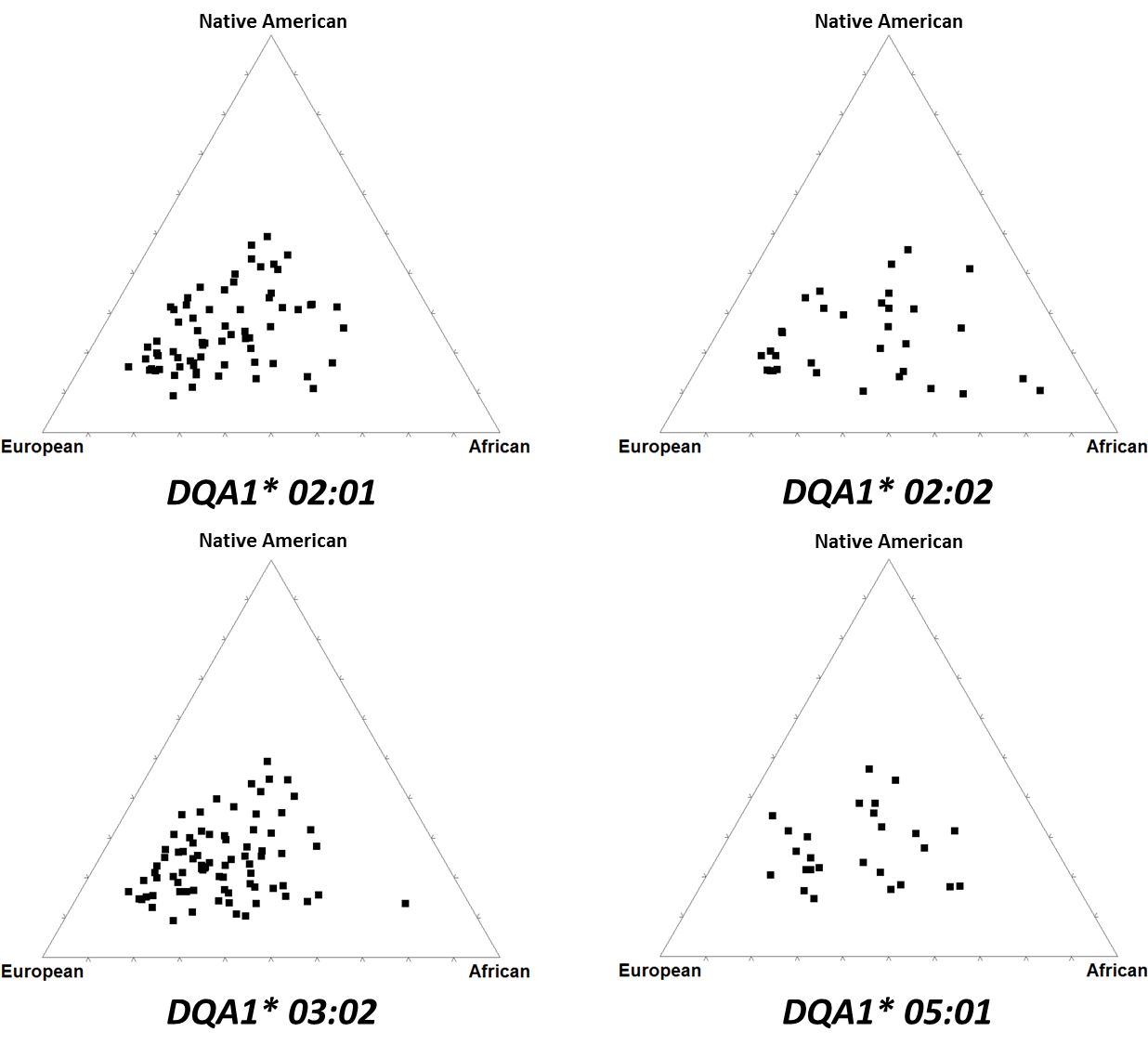
**

**Figure 4bS. Triangle plot of autosomal ancestry according to *DQA1* group.**

African (p = 0.025), Native American (p = 0.286), European (p = 0.062)

**
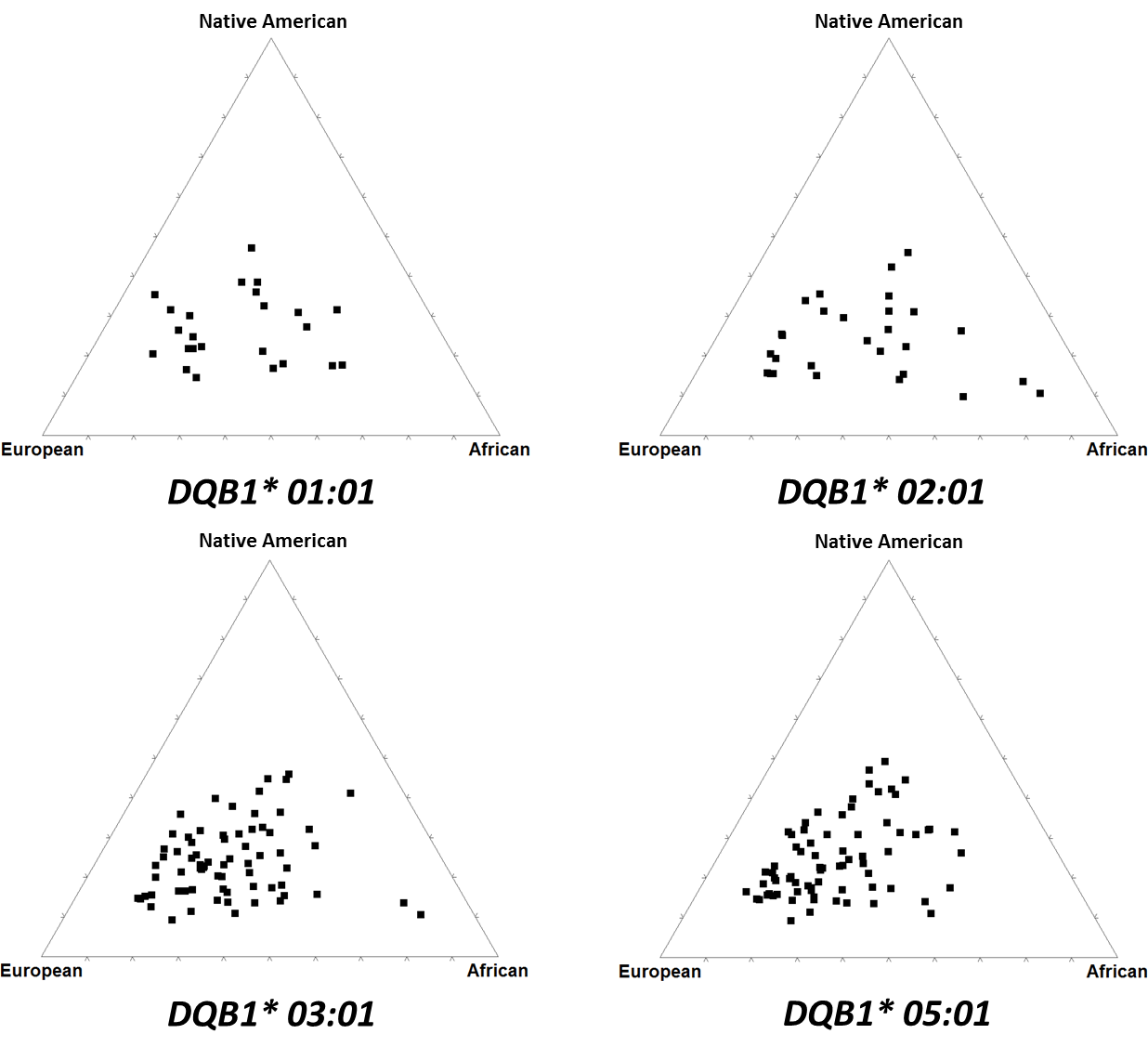
**

**Figure 4cS. Triangle plot of autosomal ancestry according to *DQB1* group.**

African (p= 0.066), Native American (p = 0.617), European (p = 0.149)
